# Enhanced Susceptibility to Spontaneous Seizures of Noda Epileptic Rats by Loss of Synaptic Zn^2+^

**DOI:** 10.1371/journal.pone.0071372

**Published:** 2013-08-12

**Authors:** Atsushi Takeda, Masashi Iida, Masaki Ando, Masatoshi Nakamura, Haruna Tamano, Naoto Oku

**Affiliations:** 1 Department of Bioorganic chemistry, School of Pharmaceutical Sciences, University of Shizuoka, Global COE-21, 52- 1 Yada, Suruga-ku, Shizuoka, Japan; 2 Department of Medical Biochemistry, School of Pharmaceutical Sciences, University of Shizuoka, Global COE-21, 52- 1 Yada, Suruga-ku, Shizuoka, Japan; The Florey Institute of Neuroscience and Mental Health, Australia

## Abstract

Zinc homeostasis in the brain is associated with the etiology and manifestation of epileptic seizures. Adult Noda epileptic rats (NER, >12-week-old) exhibit spontaneously generalized tonic-clonic convulsion about once a day. To pursue the involvement of synaptic Zn^2+^ signal in susceptibility to spontaneous seizures, in the present study, the effect of zinc chelators on epileptogenesis was examined using adult NER. Clioquinol (CQ) and TPEN are lipophilic zinc chelotors, transported into the brain and reduce the levels of synaptic Zn^2+^. The incidence of tonic-clonic convulsion was markedly increased after i.p. injection of CQ (30–100 mg/kg) and TPEN (1 mg/kg). The basal levels of extracellular Zn^2+^ measured by ZnAF-2 were decreased before tonic-clonic convulsion was induced with zinc chelators. The hippocampal electroencephalograms during CQ (30 mg/kg)-induced convulsions were similar to those during sound-induced convulsions in NER reported previously. Exocytosis of hippocampal mossy fibers, which was measured with FM4-64, was significantly increased in hippocampal slices from CQ-injected NER that did not show tonic-clonic convulsion yet. These results indicate that the abnormal excitability of mossy fibers is induced prior to epileptic seizures by injection of zinc chelators into NER. The incidence of tonic-clonic convulsion induced with CQ (30 mg/kg) was significantly reduced by co-injection with aminooxyacetic acid (5–10 mg/kg), an anticonvulsant drug enhancing GABAergic activity, which did not affect locomotor activity. The present paper demonstrates that the abnormal excitability in the brain, especially in mossy fibers, which is potentially associated with the insufficient GABAergic neuron activity, may be a factor to reduce the threshold for epileptogenesis in NER.

## Introduction

Zinc homeostasis in the brain is associated with the etiology and manifestation of epileptic seizures [1], [2]. The three synapses in the hippocampus are stained by Timm’s sulfide-silver method [3], which detects vesicular zinc to serve as a signal factor (Zn^2+^ signal). Zn^2+^ is co-released with glutamate and modulates signal transduction via glutamate at zincergic synapses [4]–[8]. Because synaptic Zn^2+^ serves as a negative feedback factor against glutamate release in the hippocampus [9], [10], it is likely that synaptic Zn^2+^ levels is involved in pathophysiology of epileptic seizures [11]–[13]. Temporal lobe epilepsy, in which seizures frequently originate in the hippocampus, causes neuronal death in the hippocampus [14]. The increase in extracellular glutamate in the hippocampus may trigger spontaneous seizures in patients with complex partial epilepsy [15]. Intracellular Zn^2+^ signal increases through Zn^2+^ influx and Zn^2+^ release from the internal stores after seizures and can be involved in neurodegeneration [16], [17].

On the other hand, zinc concentration in the brain, especially in the hippocampus, is decreased by epileptic seizures [18], [19]. Although there is no evidence that the decrease modifies susceptibility to epileptic seizures, it is possible that reduction of synaptic Zn^2+^ facilitates epileptic seizures [20]. However, there are controversial reports on the action of synaptic Zn^2+^ in epileptogenesis. In zinc transporter 3 (ZnT3) gene-null mice, a strain that lacks zinc in the synaptic vesicle, kainate induces seizures to a similar degree in wild type and ZnT3-null mice [16]. In contrast, Cole et al. [21] report that ZnT3-null mice are more susceptible than wild-type mice to limbic seizures elicited by kainate, suggesting that the net effect of hippocampal Zn^2+^ on acute seizures in vivo is inhibitory. Therefore, physiological significance of synaptic Zn^2+^ signal in epileptic seizures, especially in spontaneous seizures remains to be solved.

Spontaneous seizures contribute to understanding the nature of human epilepsy. Acute loss of Zn^2+^ signal by zinc chelators is a reasonable strategy to study the significance of synaptic Zn^2+^ signal in animal models with spontaneous seizures. It is required that zinc chelators do not interfere with the tightly bound zinc pool, such as zinc fingers and numerous catalytic enzymes, which are essential for cellular functions. Clioquinol (5-chloro-7-iodo-8-hydroxyquinoline; CQ) forms lipophilic chelates with cations such as Zn^2+^ and Cu^2+^, and is a candidate for the transient loss of Zn^2+^ signal [22], [23]. CQ has a relatively weak affinity for zinc (K_d_, approximately 1×10^−7^ M). N,N,N′,N′-Tetrakis-(2-pyridylmethyl) ethylendediamine (TPEN: K_d_ = 2.6×10^−16^ M) also forms a lipophilic chelate with Zn^2+^ and has a strong affinity for zinc [24]. The use of both chelators is a strategy to assess the action of the chelators except for zinc chelation in epileptic seizure.

Here we examined the effects of acute loss of Zn^2+^ signal on epileptogenesis in adult Noda epileptic rats (NER, >12-week-old), which show spontaneous tonic–clonic convulsion without any external stimuli once every 30 h [25]–[27]. The convulsion is characterized by the appearance of high voltage polyspikes in cortical and hippocampal electroencephalograms. On the basis of the data that zinc chelators enhanced seizure susceptibility, the effect of zinc chelation on epileptogenesis was assessed focused on the imbalance of excitation-inhibition at zincergic (mossy fiber) synapses prior to epileptic seizures.

## Results

### Enhanced Susceptibility to Spontaneous Seizures of NER by Zinc Chelators

**Figure 1:**
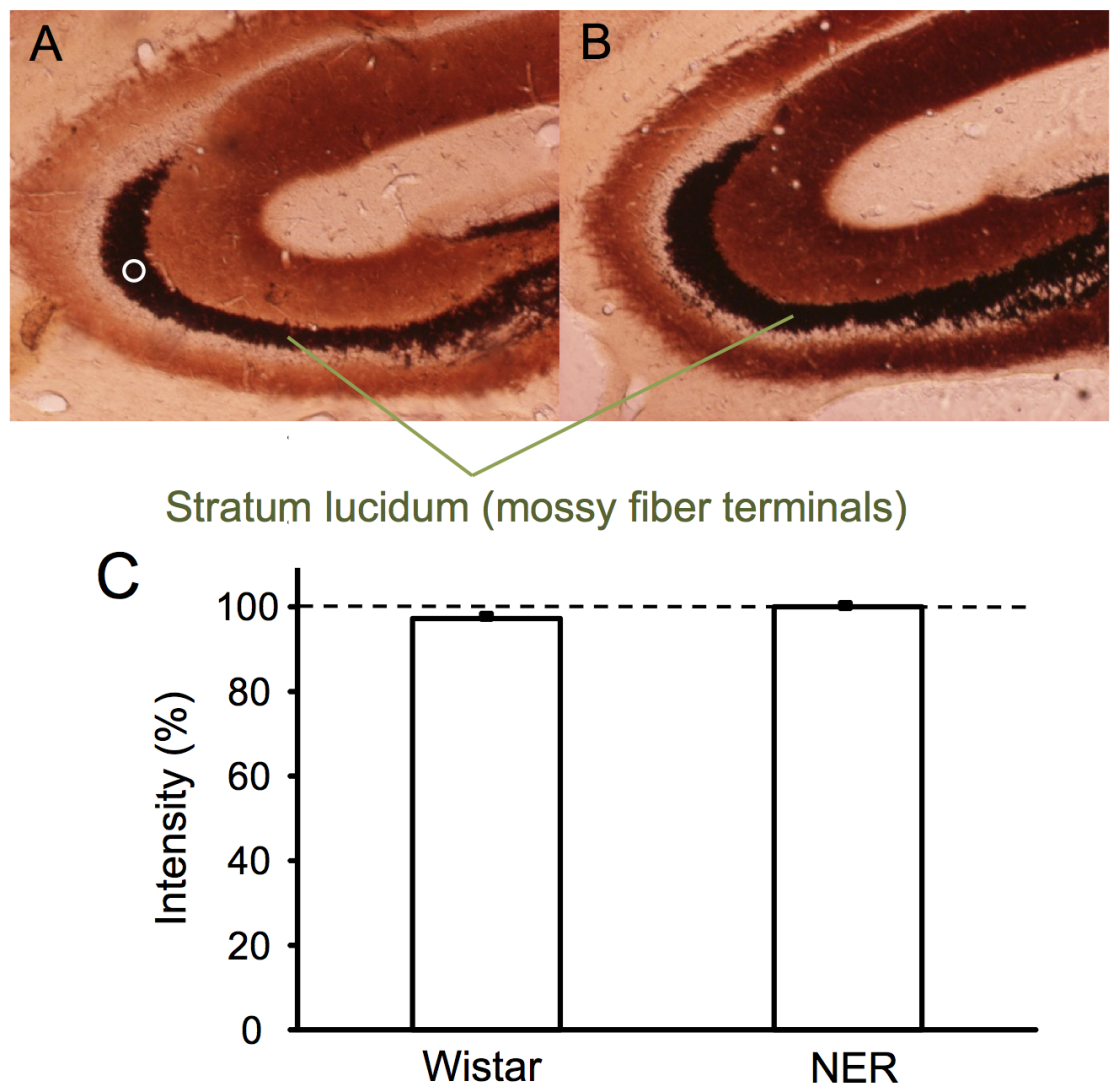
Timm’s staining in the hippocampus. Hippocampal slices were prepared from Wistar rats (A) and NER (B) (n = 10). The lower panel show the density of Timm’ stain, which was measured by using Multi Gauge V3.1, in the stratum lucidum where mossy fiber terminals exist. As a representative sample (circle) in Fig. 1A, five regions of interest per slice were set in the stratum lucidum and the densities measured were averaged. Each bar and line (mean ± SEM) represents the rate (%) of the density of Timm’ stain of Wistar rats to that of NER, which was represented as 100%.

**Figure 2:**
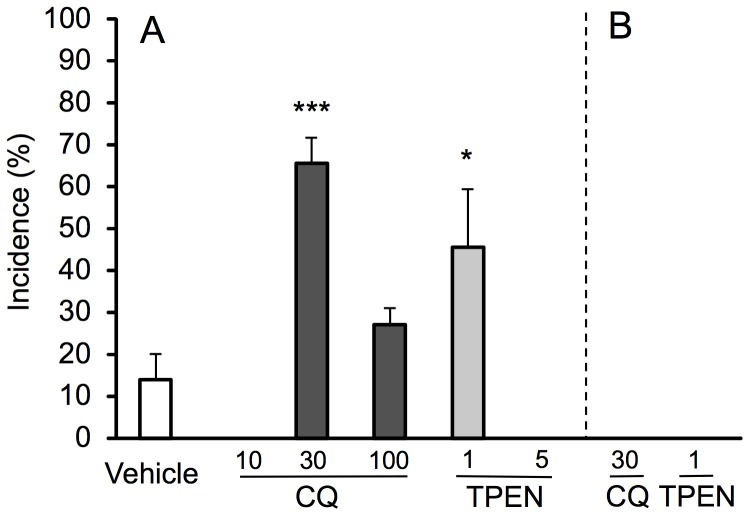
Seizure susceptibility in NER after injection of zinc chelators. NER were i.p. injected with clioquinol (10, 30, 100 mg/kg) or TPEN (1, 5 mg/kg) and then the behavior was observed for 6 h in the home cage. The incidence represents the rate of seized rats, which exhibited tonic-clonic convulsion, to the total rats (vehicle, n = 42; CQ 10 mg/kg, n = 4; CQ 30 mg/kg, n = 48; CQ 100 mg/kg, n = 19; TPEN 1 mg/kg, n = 16, TPEN 5 mg/kg, n = 8) (A). Note that no seizures were observed in Wistar rats after injection of clioquinol (30 mg/kg, n = 12) and TPEN (1 mg/kg, n = 12) (B). Each bar and line represents the mean ± SEM. *, p<0.05, ***, p<0.001, vs. vehicle.

On the basis of the findings that the hippocampus may play a role in epileptogenicity in NER [27], the levels of Zn^2+^ signal were checked in the hippocampus. The stain image of the hippocampus by Timm’s method, which assesses the levels of vesicular zinc to serve as Zn^2+^ signal, was not significantly different between the control (Wistar strain) and NER (Fig. 1A and 1B); the stratum lucidum where mossy fiber terminals from dentate granule cells exist was stained similarly between them (Fig. 1C). Adult NER exhibit spontaneously generalized tonic-clonic convulsion once every 30 h [25]. In the present study, the rate of spontaneous seizures was indicated as the incidence after vehicle injection, which is estimated to be around 20% as the probability in the observation for 6 h. The incidence of vehicle-injected NER was 13.2±6.3% (Fig. 2A). Furthermore, the effect of zinc chelators on epileptogenesis in NER was examined to pursue the involvement of synaptic Zn^2+^ signal. The incidence of tonic-clonic convulsion was markedly increased after intraperitoneally (i.p.) injection of CQ (30–100 mg/kg) and TPEN (1 mg/kg) (Fig. 2A). The latency to tonic-clonic convulsion was 2.9±0.3 h after CQ (30 mg/kg) injection and 3.5±1.0 h after TPEN (1 mg/kg) injection. TPEN (5 mg/kg) significantly reduced locomoter activity and did not show tonic-clonic convulsion. In NER that showed tonic-clonic convulsion, status epilepticus was observed after injection of CQ (30 mg/kg, 38.5%; 100 mg/kg, 37.5%) and TPEN (1 mg/kg, 25.0%), but not after injection of vehicle. On the other hand, no seizures were observed in Wistar rats (twelve per each group) after i.p. injection of clioquinol (30 mg/kg) and TPEN (1 mg/kg) (Fig. 2B).

**Figure 3:**
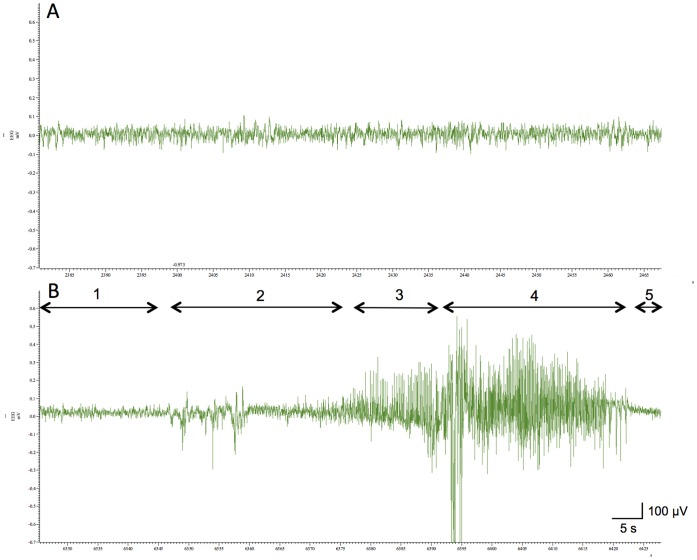
Ictal electroencephalogram recorded in CQ-injected NER. A recording electrode was implanted into the hippocamal CA3 region of NER. Two days later, vehicle (A) or clioquinol (30 mg/kg) in vehicle (B) was i.p. injected into NER and then the behavior was observed in the home cage until seizures were observed. Note that no seizure was observed in vehicle-treated NER. The basal activity before seizures (1) and the stages of seizures were observed as follows: beginning of aberrant activity on electroencephalogram without behavioral changes (2); asymmetric wave with myoclonus (3); high-voltage wave with tonic-clonic convulsion (4); low-voltage wave with postictal flaccidness (5). The component of γ-wave (7–9 Hz) (2, 6.9%; 3, 19.4%; 4, 15.4%; 5, 10.0%) became rhythmic during seizures as reported previously [26].

The hippocampal electroencephalogram during CQ-induced tonic-clonic convulsion is shown in Fig.3. Aberrant activity started with small spikes superimposed on irregular spikes. The rat showed sudden prodromal signs of convulsion, e.g., myoclonus, in association with asymmetric spikes. These spikes increased in amplitude and frequency and become rhythmic at a frequency of 7–9 Hz, concurrent with the appearance of opisthotonic posture and followed by tonic-clonic convulsion.

### Enhancement of Presynaptic Activity by Reduction of Zn^2+^ Signal

**Figure 4:**
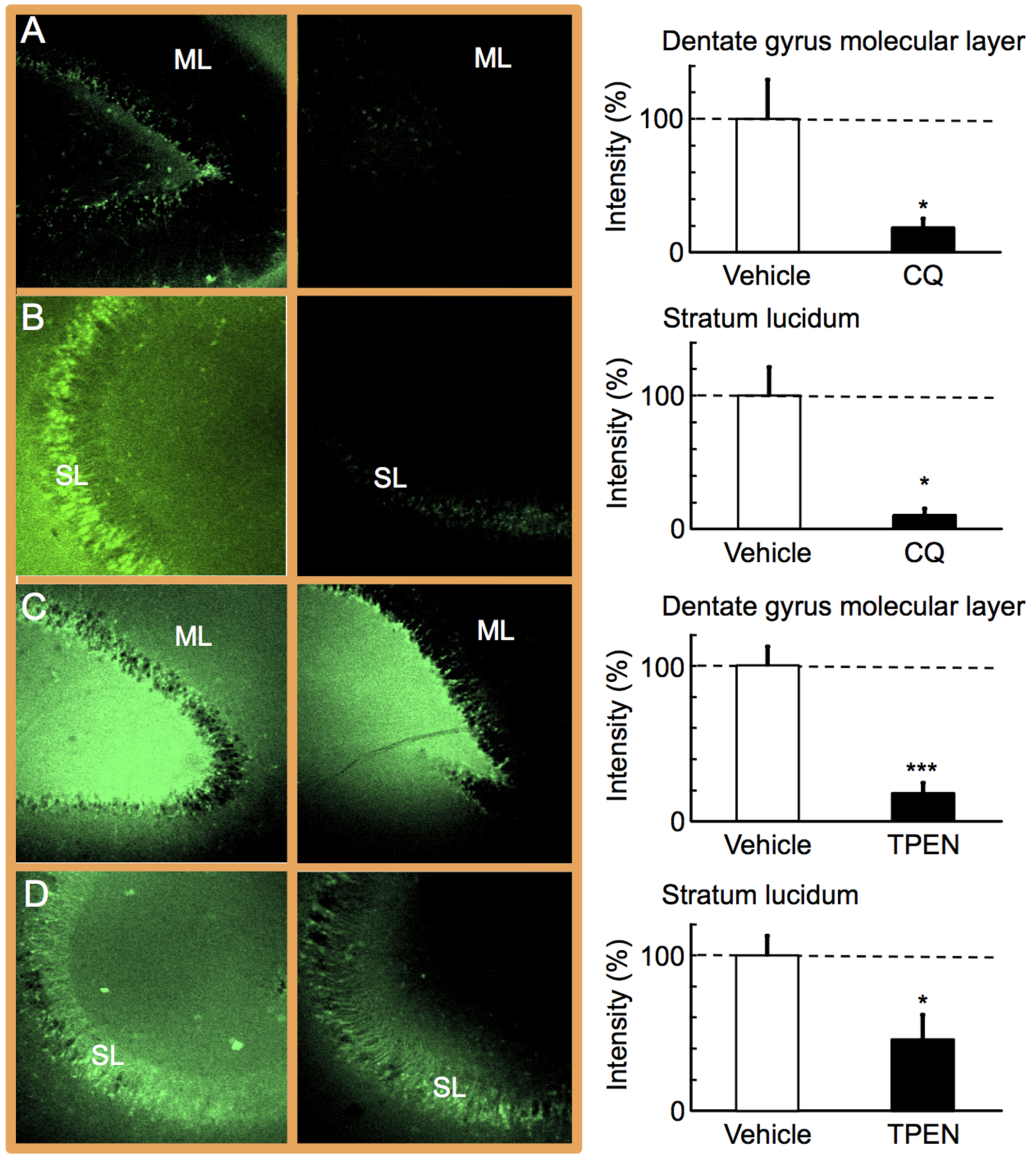
Decrease in extracellular Zn^2+^ in the hippocampus by zinc chelators. Hippocampal slices were prepared 2 h after i.p. injection of vehicle (A, n = 7) or CQ (30 mg/kg, B, n = 5) into NER, and 3 h after i.p. injection of vehicle (C, n = 7) or TPEN (1 mg/kg, D, n = 8) into NER. The hippocampal slices were imaged with ZnAF-2 (left side). To evaluate the effects of CQ (the left image of A and B, vehicle; the right image of A and B, CQ) and TPEN (he left image of C and D, vehicle; the right image of C and D, TPEN), CQ-injected (A and B) and TPEN-injected (C and D) groups were separately measured under the exactly same condition. The measurement condition was slightly different between CQ and TPEN groups for the optimal imaging of the decrease in Zn^2+^ by fluorescence intensity. Region of interest was set the area surrounding the abbreviated letters, i.e., ML and SL, where zincergic (glutamatergic) synapses exist. ML, the molecular layer of the dentate gyrus (A and C). SL, the stratum lucidum of the CA3 (B and D). The data (mean ± SEM) represent the rate of ZnAF-2 intensity after zinc chelator injection to that after vehicle injection, which was expressed as 100% (right side).

On the basis of the latent period to tonic-clonic convulsion induced by zinc chelators, extracellular Zn^2+^ levels were checked with ZnAF-2 2 h and 3 h after injection of CQ and TPEN, respectively when tonic-clonic convulsion was not observed yet (Fig. 4). Both chelators markedly reduced extracellular Zn^2+^ levels in the molecular layer of the dentate gyrus and the stratum lucidum of the CA3 where zincergic synapses exist.

**Figure 5:**
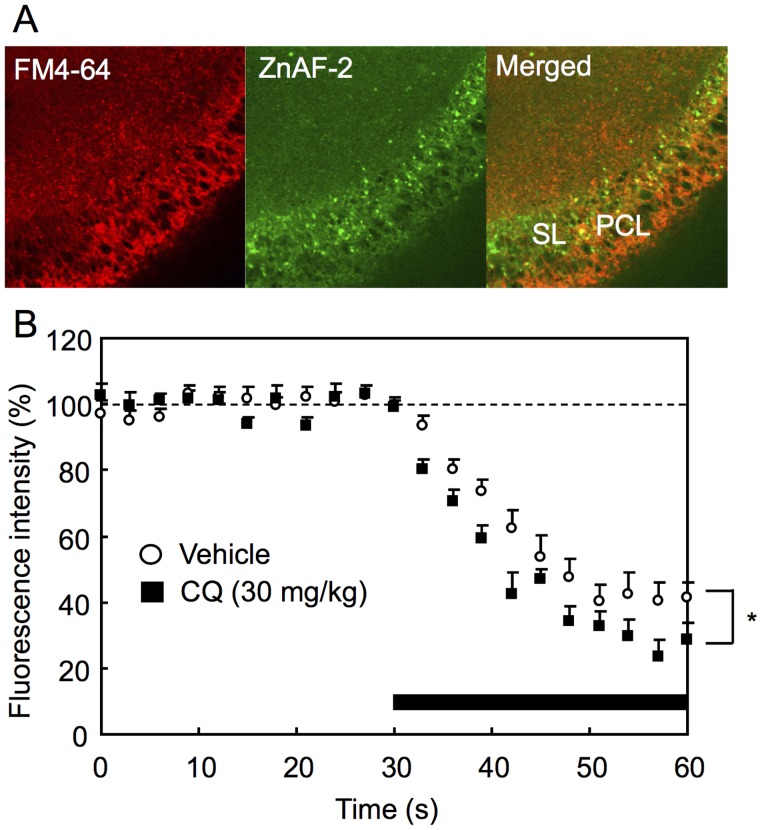
Enhanced exocytosis in mossy fibers after CQ injection. Hippocampal slices (400- µm thickness) were prepared 2 h after i.p. injection of CQ (30 mg/kg). To measure the decrease in FM4-64 fluorescence intensity in mossy fiber terminals (boutons), mossy fiber terminals were doubly stained with ZnAF-2 and FM4-64. ZnAF-2 staining was done under an optimal condition to stain mossy fiber terminals clearly, unlike the ZnAF-2 staining condition of Fig. 4. The terminals strongly stained with ZnAF-2 were determined as region of interest. To observe presynaptic activity, tetanic stimulation at 10 Hz for 30 s, which was shown by a shaded bar, was delivered to mossy fibers and then single strong stimulation at 100 Hz for 18 s was delivered to the same position. The activity-dependent component of FM4-64 signal was measured for each punctum (1 s) by subtracting its residual fluorescence intensity measured by the strong electrical stimulation. The data (mean ± SEM) represent the ratio (%) of each FM4-64 intensity to the basal FM4-64 intensity before tetanic stimulation at 10 Hz for 30 s, which was averaged and expressed as 100% (vehicle, n = 10; CQ, n = 12). SL, stratum lucidum; PCL, CA3 pyramidal cell layer. *, p<0.05, vs. vehicle.

To examine whether the loss of synaptic Zn^2+^ modifies exocytosis of mossy fiber terminals, mossy fiber terminals were double-stained with ZnAF-2 and FM4-64 (Fig. 5A). CA3 pyramidal cells were scarcely stained with FM4-64 and ZnAF-2 and mossy fiber synapses that innervate CA3 pyramidal cells were strongly stained with ZnAF-2. Mossy fiber terminals, which are giant boutons, were often imaged as puncta with ZnAF-2. FM4-64 is taken up into presynaptic vesicles in an activity-dependent manner. Subsequent rounds of exocytosis arising from depolarization lead to the release of the dye from the presynaptic terminals (destaining). Because fluorescence signal originates from vesicular membrane-bound FM4-64, the fluorescence signal is attenuated by presynaptic activity [10]. Mossy fiber activity was evaluated by attenuation of FM4-64 fluorescence during the tetanic stimulation (Fig. 5B). Attenuation of FM4-64 fluorescence in mossy fiber terminals was significantly enhanced in the hippocampal slices prepared 2 h after CQ (30 mg/kg) injection when tonic-clonic convulsion was not observed yet.

### Suppression of Zinc Chelator-induced Seizures by Enhancing GABAergic Activity

**Figure 6:**
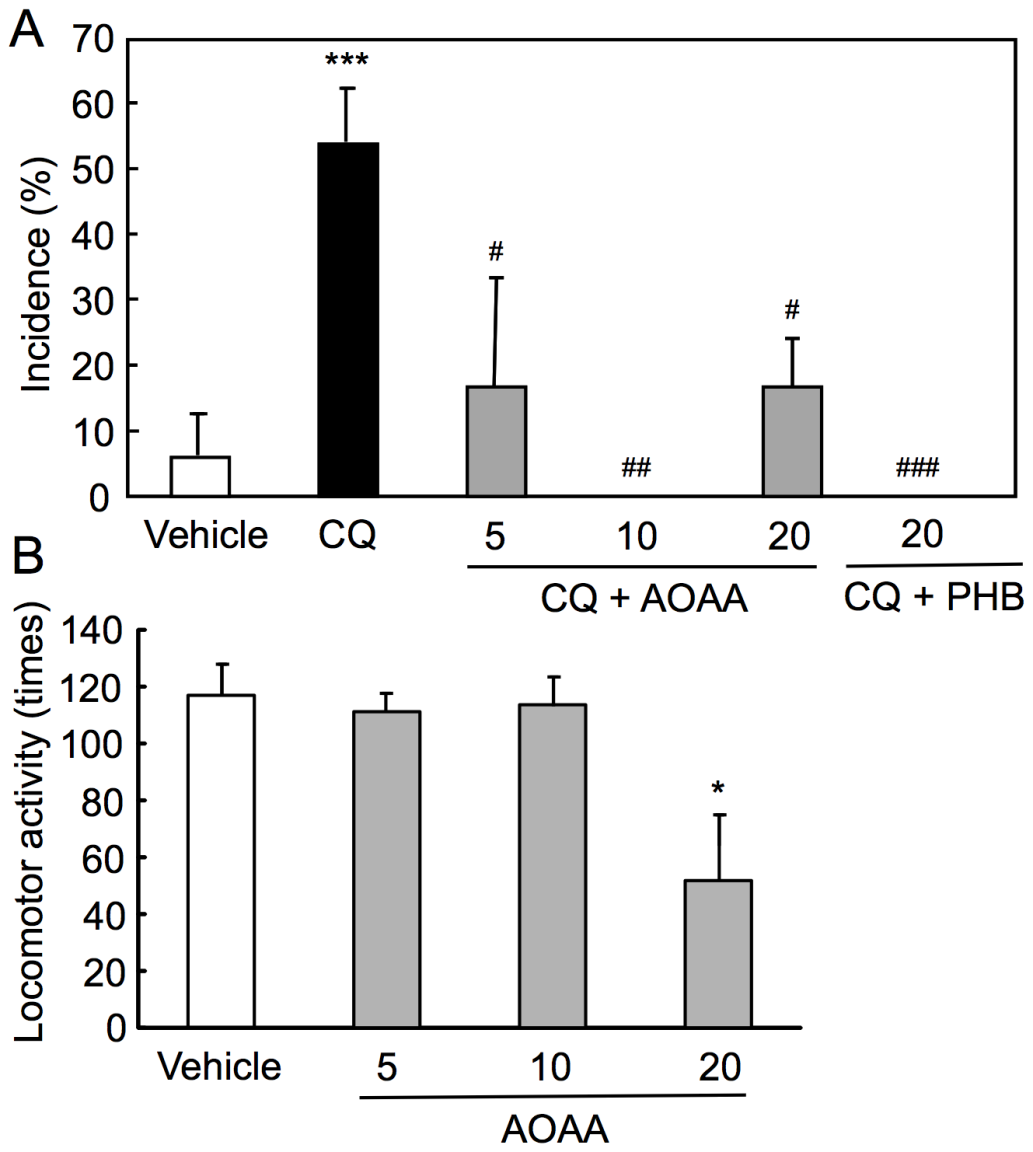
Suppression of CQ-induced seizures in NER by enhancing GABAergic activity. AOAA (5, 10, 20 mg/kg) or phenobarbital (PHB, 20 mg/kg) was i.p. co-injected with CQ (30 mg/kg) into NER, and then the behavior was observed for 6 h in home cage. The incidence represents the rate of seized rats, which exhibited tonic-clonic convulsion, to the total rats (vehicle, n = 22; CQ 30 mg/kg, n = 38; CQ+AOAA (5 mg/kg), n = 10; CQ+AOAA (10 mg/kg), n = 10; CQ+AOAA (20 mg/kg), n = 20; CQ+PHB (20 mg/kg), n = 18). Each bar and line represents the mean ± SEM (A). ***, p<0.001, vs. vehicle; ^#^, p<0.05, ^##^, p<0.01, ^###^, p<0.001, vs. CQ. To check the effect of AOAA on locomotor activity, NER were subjected to the open-field test 2 h after i.p. injection of AOAA (n = 10). Each bar and line represents the mean ± SEM (B). *, p<0.05, vs. vehicle.

To examine the effect of drugs enhancing GABAergic activity on zinc chelator-induced seizures, aminooxyacetic acid(AOAA, 5–20 mg/kg), a GABA transaminase inhibitor and phenobarbital (20 mg/kg), a potentiator of GABA_A_ receptors were co-injected with CQ (30 mg/kg) into NER. Both drugs significantly reduced the incidence of tonic-clonic convulsion (Fig. 6A). The averaged latency of CQ-induced seizures was longer after co-injection of AOAA (CQ, 2.9 h; CQ+AOAA (5 mg/kg), 3.8 h; CQ+AOAA (20 mg/kg), 3.2 h). Because AOAA (20 mg/kg) and phenobarbital seemed to affect locomotor activity, the action of these drugs in locomotor activity was checked in the open-field test. Administration of AOAA (5–10 mg/kg), unlike the dose (20 mg/kg), did not affect locomotor activity of NER (Fig. 6B) and administration of phenobarbital (20 mg/kg) reduced locomotor activity of NER (data not shown).

## Discussion

Zinc has been proven to be anticonvulsant in several studies [28], which indicate that diphenylthiocarbazone (dithizone) and diethyldithiocarbamate (DEDTC), zinc chelators, enhance seizure activities [29], [30]. In the amygdala rapid kindling model, on the other hand, DEDTC decreased the duration of both behavioral seizures and electrical afterdischarges, and also decreased the electroencephalogram spike frequency, without changing the progression of behavioral seizure severity, suggesting that synaptic Zn^2+^ has a facilitatory role during kindling progression [31]. Lavoie et al. [32] report that intracellular but not extracellular zinc chelators influence neuronal excitability of the CA3 region using a high [K^+^]_o_ model of epilepsy for status epilepticus. On the basis of the controversial evidence on the action of synaptic Zn^2+^ in experimentally induced seizures, in the present study, we used NER, a genetic model in epilepsy research to understand synaptic Zn^2+^ function in susceptibility to spontaneous seizures. Adult NER frequently exhibit spontaneous convulsions, controlled by a major autosomal recessive gene for epilepsy, that are comparable to generalized tonic-clonic seizures in humans [26].

The incidence of tonic-clonic convulsion was markedly increased in NER after i.p. injection of CQ (30 mg/kg) and TPEN (1 mg/kg), which did not induce any convulsion in the control rats (Wistar strain). Both CQ and TPEN decrease intracellular Zn^2+^ levels [22], [24]. The basal levels of extracellular Zn^2+^ measured by ZnAF-2 were decreased before tonic-clonic convulsion was induced with zinc chelators. It is well known that epileptic seizures are closely linked to external stimuli in human. External stimui have been usually used for the analysis on tonic-clonic activity in spontaneous epilepsy animals [26], [33]. The hippocampal electroencephalogram during CQ (30 mg/kg)-induced convulsion was similar to that during sound-induced convulsions in NER reported previously [26]. These results suggest that the loss of synaptic Zn^2+^ signal with zinc chelators is a factor to induce epileptic seizures in NER. On the other hand, abnormal excitation in epileptic seizures reduces synaptic Zn^2+^ levels, as well as the total zinc in the brain [19]. It is likely that this reduction may also enhance seizure susceptibility as well as the action of zinc chelators. The data that status epilepticus was frequently observed in NER after injection of zinc chelators, unlike the control (vehicle-injected rats) is consistent with the idea. In the present study, two zinc chelators were used to access the actions except for zinc chelation. TPEN has a strong affinity for zinc and the chelation of Zn^2+^ from proteins with TPEN may reduce cell viability [24]. The dose (5 mg/kg) of TPEN reduced locomotor activity and did not induce any convulsion. The data that vehicle-injected NER showed tonic-clonic convulsion at a low rate proposes an idea that neurotoxicity of lipophilic zinc chelator may influence seizure susceptibility.

Kiura et al. [34] report that calcium channel dysfunction may be involved in the abnormal excitability of CA3 pyramidal neurons and pathogenesis of epilepsy in NER. Glutamatergic mossy fibers innervate CA3 pyramidal neurons and GABAergic interneurons. Zn^2+^ released from mossy fibers may deceases glutamate release from mossy fibers as a negative feedback factor [9], [10] and may enhance GABAergic neurotransmission system under excessive excitation [35]. It is reported that Zn^2+^ inhibits GABA transporter 4, which is highly expressed in the cerebral cortex and hippocampal CA3 and CA1, and may suppress glutamate-induced excitotoxicity [36]. Thus, it is possible that the loss of synaptic Zn^2+^ with zinc chelators induces the imbalance of excitation-inhibition at mossy fiber-CA3 pyramidal neuron synapses. Exocytosis of mossy fibers from dentate granule cells, which measured with FM4-64, was significantly increased in hippocampal slices prepared 2 h after CQ injection when tonic-clonic convulsion was not observed yet. The present paper is the first to demonstrate that the abnormal excitability of mossy fibers is induced prior to epileptic seizures by injection of zinc chelators into NER. However, zinc chelators act on the whole brain and nonspecifically reduce synaptic Zn^2+^ in the brain. It is possible that the changes in other brain regions except for the hippocampus are required for epileptogenesis.

Any drug that has an anticonvulsant action can suppress seizures. The drugs often affect locomotor activity and induce immobilized and/or sleepy situations. If seizure susceptibility is linked to abnormal glutamatergic neuron activity, excess of GABAergic neuron activity may be required to suppress epileptic seizures, followed by the affected locomotor activity with the drugs. The locomotor activity is an index to assess physiological brain function. The doses (5–10 mg/kg) of AOAA that did not affect the locomotor activity suppressed epileptic seizures, suggesting that the insufficient GABAergic neuron activity is associated with abnormal glutamatergic neuron activity.

In conclusion, seizure susceptibility was enhanced selectively in NER after administration of zinc chelators. This selectivity is an important evidence to understand the epileptogenesis in NER. On the other hand, the imbalance of excitation and inhibition, i.e., the abnormal glutamatergic neuron activity can be induced even in the control rats treated with zinc chelator [10], [20], [35]. Therefore, it is possible that the extent of excessive glutamatergic neuron activity through the loss of synaptic Zn^2+^ with zinc chelators is different between wild type (Wistar) rat and NER. Knock-out of zinc transporters Zip-1 and Zip-3 exhibits a lower seizure threshold to kainate, suggesting that Zn^2+^ dyshomeostasis via the zinc transporters is involved in seizure severity induced with kainate [37]. The present paper demonstrates that the abnormal excitability in the brain, especially in mossy fibers, which is potentially associated with the insufficient GABAergic neuron activity, may be a factor to reduce the threshold for epileptogenesis in NER. The trigger (cause) to elicit epileptogenesis remains to be clarified.

## Materials and Methods

### Animals and Chemicals

Male Noda epileptic rats (15–20 weeks old) were kindly supplied from Japan SLC (Hamamatsu, Japan). Male Wistar rats (15 weeks old) were purchased from Japan SLC. Rats were housed under the standard laboratory conditions (23±1°C, 55±5% humidity) and had access to tap water and food ad libitum.

CQ and TPEN were dissolved in 20% dimethyl sulfoxide (DMSO) in olive oil. ZnAF-2, a membrane-impermeable zinc indicator, was kindly supplied from Sekisui Medical Co., LTD (Tokai, Japan), dissolved in DMSO, and then diluted to artificial cerebrospinal fluid (ACSF) containing 119 mM NaCl, 2.5 mM KCl, 1.3 mM MgSO_4_, 1.0 mM NaH_2_PO_4_, 2.5 mM CaCl_2_, 26.2 mM NaHCO_3_, and 11 mM D-glucose (pH 7.3). ZnAF-2 has a low K_d_ value of 2.7 nM for zinc [38].

### Ethics Statement

All experiments were performed in accordance with the Guidelines for the Care and Use of Laboratory Animals of the University of Shizuoka that refer to American Association for Laboratory Animals Science and the guidelines laid down by the NIH (*NIH Guide for the Care and Use of Laboratory Animals*) in the USA. The Animal Experiment Committee of University of Shizuoka approved all protocols for animal experiments (#24–017).

### Timm’s Sulfide-silver Staining

Rats were deeply anesthetized with chloral hydrate and then perfused transcardially with 0.1% Na_2_S in phosphate buffer (pH 7.4). The brains were excised and immersed in 4% (w/v) paraformaldehyde in 0.1 M sodium phosphate buffer (pH 7.4) for 24 h and then in 10–30% sucrose for 72 h. Coronal 30 µm sections were prepared in a cryostat at −20°C. Timm’s staining was performed according to the procedure described previously [39]. The relative density was measured with a microscope (OLYMPUS IX71; software, Multi Gauge V3.1) [40]. Although mossy fiber sprouting is often observed in the hippocampus of epileptic animal models [41], the morphology of the mossy fibers with Timm’s stain was not appreciably different between the control and NER. As a representative sample (circle) in Fig. 1A, five regions of interest per slice were set in the stratum lucidum of the CA3 where mossy fiber terminals exist and the densities measured were averaged. Timm’s density in the stratum lucidum of NER was normalized as 100% and Timm’s density in the stratum lucidum of Wistar rats was represented as the rate (%) to that of NER. Mossy fiber terminals are observed at the highest density in the hippocampus of both rats.

### Seizures After Injection of Zinc Chelators and Drugs

The behavior of rats was recorded for 6 h with a video camera after rats were i.p. injected with zinc chelators and drugs. When seizures are induced in NER, tonic-clonic convulsion was always observed in all rats tested. Tonic-clonic convulsion was used as the incidence of epileptic seizures.

### Open Field

Locomotor activity of rats was assessed in the open-field test. Each rat was placed in an arena (70×70×64 cm) made of a black-colored wooden box, in which it has never been placed. Behavior of each rat in the arena was recorded with a video camera and locomotor activity of rats was measured for 5 minutes.

### Hippocampal Electroencephalograms

Male rats were anesthetized with chloral hydrate (400 mg/kg) and placed in a stereotaxic apparatus. A monopolar recording electrode made of tungsten wire was positioned stereotaxically so as to selectively record in the left CA3; the recording electrode was implanted ipsilaterally 5.6 mm posterior to the bregma, 4.6 mm lateral and 6.2 mm inferior to the dura. An indifferent electrode was positioned on the cranium. Two days later, rats were i.p. injected with CQ (30 mg/kg) in vehicle and placed in a polystyrene box (diameter, 32 cm; depth, 30 cm) set in a shielded chamber. The behavior of rats and electroencephalograms were simultaneously recorded using software (The Spike2, Cambridge Electronic Design Limited) after injection of CQ.

### Hippocampal Slice Preparation

Rats were anesthetized with ether and decapitated. The brain was quickly removed and immersed in ice-cold ACSF. Transverse hippocampal slices (400 µm) were prepared using a vibratome ZERO-1 (Dosaka Kyoto, Japan) in an ice-cold ACSF. Slices were then maintained in a holding chamber at room temperature for at least 1 h. All solutions used in the experiments were continuously bubbled with 95% O_2_ and 5% CO_2_.

### Extracellular Zinc Imaging

The hippocampal slices were transferred to a recording chamber filled with 10 µM ZnAF-2 in ACSF. The fluorescence of ZnAF-2 (excitation, 488 nm; monitoring, 505–530 nm) was measured in the hippocampus by using a confocal laser-scanning microscopic system LSM 510 (Carl Zeiss), equipped with the inverted microscope (Axiovert 200M, Carl Zeiss). Region of interest was set in the molecular layer of the dentate gyrus and the stratum lucidum of the CA3.

### Mossy Fiber Activity (Exocytosis)

The presynaptic activity was measured by using FM4-64 according to the previous paper [42], [43]. The hippocampal slices were transferred to an incubation chamber filled with ACSF containing 5 µM FM4-64, an indicator of presynaptic activity and 45 mM KCl, allowed to stand at 25°C for 90 s, transferred a chamber filled with ACSF to wash out extracellular FM4-64, and transferred to a recording chamber filled with 10 µM ZnAF-2 in ACSF containing 10 µM 6-cyano-7-nitroquinoxaline-2,3-dione (CNQX), an antagonist of AMPA/kainate receptors to prevent recurrent activity. The basal fluorescence of FM 4-64 (excitation, 488 nm; monitoring, above 650 nm) and ZnAF-2 (excitation, 488 nm; monitoring at 505–530 nm) were measured for 30 s with a confocal laser-scanning microscopic system LSM 510 META. Electrical stimuli (10 Hz, 30 sec, 100 µA, 200 µs/pulse) were delivered to the dentate granular cell layer through a bipolar tungsten electrode. The fluorescence of FM 4–64 and ZnAF-2 were measured in the same manner to observe the attenuation of FM 4–64 fluorescence (destaining) based on presynaptic activity. At the end of the experiments, complete depolarization-induced destaining was evoked by single strong stimuli (100 Hz, 18 s, 100 µA, 200 µs/pulse). The activity-dependent component of FM4-64 fluorescence in the stratum lucidum, where ZnAF-2 fluorescence was increased, was measured for each punctum by subtracting its residual fluorescence intensity (<10% of initial intensity) measured after the strong electrical stimulation that produced maximal destaining. FM4-64 signal was then normalized by the maximal fluorescence intensity before the electrical stimulation.

### Statistical Analysis

Grouped data are expressed as the mean ± SEM. Student’s *t*-test was used for comparison of the means of paired or unpaired data. For multiple comparisons, two-way ANOVA was used as indicated (the statistical software, GraphPad Prism 5).
